# Risk Assessment and Management Strategies for Odor Release During the Emergency Excavation of VOC-Contaminated Wastes

**DOI:** 10.3390/toxics13060457

**Published:** 2025-05-30

**Authors:** Xiaowei Xu, Jun Zhang, Yi Wang, Haifeng Tu, Yang Lv, Zehua Zhao, Dapeng Zhang, Qi Yu

**Affiliations:** 1 Nanjing Institute of Environmental Science, Ministry of Ecology and Environment of China, Nanjing 210042, China; xuxiaowei@nies.org (X.X.); zhangjungf@163.com (J.Z.); wangyi@nies.org (Y.W.); tuhaifeng@nies.org (H.T.); lvyang@nies.org (Y.L.); 2 Department of Environmental Science, School of Environmental Science and Engineering, Suzhou University of Science and Technology, Suzhou 215009, China; yuqi@usts.edu.cn

**Keywords:** VOCs, odor risk, health impact, management strategies

## Abstract

This study examines the assessment and management strategies for odor risks during emergency cleanup of VOC-contaminated waste. By analyzing illegally dumped VOC waste, the impact on odor intensity levels and exceedance probabilities in nearby residential areas was evaluated. Utilizing a VOC source emission model, a Gaussian plume dispersion model, and Monte Carlo simulations under various meteorological conditions, the effectiveness of the control measures was assessed. Key pollutants included ethylbenzene, toluene, styrene, and m/p-xylene, which, despite posing minimal short-term health risks (PHI: 0.17–0.64), exhibited significant odor risks (Odor PHI: 127–1156). At 20 m from the source, the probability of the odor intensity exceeding Level 2.5 approached 100%, decreasing to 85% at 50 m and further declining with distance. Atmospheric stability shifts—from very unstable (Class A) to stable (Class F)—increased the odor intensity from 0.5 to 2.5. Under moderately stable conditions (Class E), m/p-xylene had a 44.2% probability of exceeding an odor intensity level of 2.5. Even at 250 m, the odor intensity levels ranged between 1.2 and 1.7, remaining perceptible. Effective mitigation strategies include establishing appropriate buffer distances and using adsorption materials like activated carbon.

## 1. Introduction

Since the release of the “Notice on Severely Combating Environmental Illegal and Criminal Behaviors of Hazardous Wastes” [1], the Ministry of Ecology and Environment has carried out targeted campaigns against environmental violations, particularly the illegal transfer, dumping, and disposal of hazardous wastes. Over 20,000 related cases have been investigated and resolved, effectively alleviating the serious pressure caused by such illegal activities [1]. However, current management practices primarily focus on the impacts of hazardous wastes on soil and groundwater, while the odor risk associated with volatile organic compound (VOC)-contaminated waste has been largely overlooked.

During emergency clean-up operations, the extent of illegal dumping or landfilling of VOC-containing waste is often found to be extensive. The escape of VOCs from waste materials may lead to odor concentrations in surrounding residential areas that exceed the relevant environmental standards, presenting health risks to residents [2]. Yan et al. [3] investigated the influence of excavation disturbance on the levels of hexachlorocyclohexane and DDT in the air of contaminated sites. With high levels of excavation disturbance, the mass concentration of organochlorine pesticides increased in the air of both the abandoned pesticide sites and the surrounding areas. Zhang et al. [4] conducted an odor risk assessment of the excavation of VOC-contaminated soils. The results showed that the distance between the sensitive target and the contaminated site was the most important factor influencing the odor concentration at the exposure point. Overall, these models combine theoretical simulations with field monitoring and optimize parameters to enhance assessment practicality.

In the practice of assessing the risks of VOCs in contaminated sites, China mainly relies on the model recommended in the “Technical Guidelines for Risk Assessment of Soil Pollution in Construction Land” (HJ 25.3-2019) [5]. This model simulates the process of VOCs volatilizing from soil to form soil gas, further migrating to indoor and outdoor air, and the potential health risks to humans. During this assessment process, it is necessary to fully consider the uncertainties of the model’s parameters, including soil properties, meteorological conditions, building structures, and exposure parameters, among others [6]. Li et al. [7] evaluated the risk of volatile organic pollutants in landfills based on probability analysis. The results showed that for individual carcinogenic risks, only ethylbenzene, benzene, chloroform, and 1,2-dichloroethane presented a slight or moderate risk at extreme concentrations. The probability of such risks occurring ranged from 0.1% to 1%. Ji Hoon Seo et al. [8] found that exposure to VOCs can lead to health issues, with 48% of workers reporting health changes, 24% experiencing dry skin, 22% feeling fatigued, and 20% suffering from skin or eye discomfort—all closely related to VOCs exposure.

The odor risk assessment process is typically based on the olfactory effect and employs a simplified steady-state approach to evaluate risk levels. However, this method is limited in its ability to capture the non-steady-state release of pollutants and the dynamic processes governing their migration and dispersion [9], which can potentially lead to the underestimation or neglect of peak risk events. Furthermore, the commonly used steady-state assessment often relies on standardized national parameters, which fail to account for variations in risk caused by differing meteorological conditions.

This study focused on an emergency clean-up in a case involving the illegal dumping of VOC-contaminated waste. It investigated the impact of volatile organic pollutants on the intensity of the odor concentration and the probability of exceeding environmental standards in downwind residential areas. This study combined a VOC source release model, the Gaussian plume diffusion model, and the Monte Carlo simulation method. By considering different meteorological conditions, this study developed practical control strategies for effectively managing and mitigating odor-related issues in residential areas.

## 2. Materials and Methods

### 2.1. Sample Preparation and Analysis

This study examined an emergency excavation at a site with over 27,000 metric tons of VOC-contaminated waste, containing pollutants such as benzene derivatives and polycyclic aromatic hydrocarbons (PAHs). The cleanup operation lasted 25 days, during which nearby residents 50 m west reported noticeable odors.

Samples were collected following the site selection and sampling methodologies prescribed by the “Technical specification for identification of hazardous wastes” (HJ 298-2019) [10] standard, which resulted in a total of 100 samples. These samples were then quantitatively analyzed according to the procedures outlined in the “Determination of volatile organic compounds in soils and sediments: purge and trap/gas chromatography-mass spectrometry method” (HJ 605-2011) [11] standard.

### 2.2. Characterization of Pollutant Emissions

During the emergency excavation of solid waste, a conceptual model of “solid waste excavation—VOCs emission—atmospheric migration—olfactory effect” was established. Mechanical disturbance caused the VOCs’ release through gas exchange between pore gases and the atmosphere. The emitted VOCs rapidly increased the local pollutant concentrations and dispersed under air currents and wind, potentially leading to olfactory irritation in nearby populations. China’s current “Technical Guidance for Risk Assessment of Soil Pollution in Construction Land” (HJ 25.3-2019) [5] uses a linear partitioning model to calculate the VOC concentrations in pore gas [12,13], which serves to assess the risk of vapor intrusion. This model assumes full reversible equilibrium among three phases and instantaneous balance, with VOC pore gas concentration calculated using Equation (1). For a conservative assessment, it is assumed that during mechanical excavation and transfer, all VOCs in the pore gas are completely released into the surrounding air. The release rate is therefore estimated using Equation (2).

(1) $$C_{air}=C_{s}\times \frac{H\times \rho }{\theta_{w}+\rho \times K_{oc}\times f_{oc}+H\times \theta_{air}}$$

(2) $$ER=V\times N\times \theta_{air}\times C_{air}$$

where $C_{air}$ denotes the VOC concentration in the pore gas of the excavation area, measured in mg/m^3^; ER represents the VOC volatilization rate in the excavation area, expressed in mg/s; $C_{s}$ is the VOC content in the VOC-contaminated waste of the excavation area, quantified in mg/kg; *H* is the Henry’s Law constant, which is dimensionless; $K_{OC}$ is the organic carbon normalized partition coefficient with units of cm^3^/g; ρ is the bulk density of the solid waste given in g/cm^3^; $\theta_{w}$ is the volumetric fraction of the pore water; $\theta_{air}$ is the volumetric fraction of the pore gas; $f_{oc}$ is the organic carbon content, measured in g/kg; V is the digging rate of a single excavator, set to 150 m^3^/h; and N is the number of excavators operating simultaneously.

### 2.3. Characterization of Pollutant Transport and Dispersion

The models currently used to evaluate the dispersion of atmospheric pollutants include the Gaussian plume model, the AERMOD model, and the ADMS model [14,15]. Among these, the Gaussian plume model is noted for its simplicity in computation and has been extensively validated by a large body of empirical data. It has therefore become the most widely applied model. This model posits that atmospheric pollutants exhibit a Gaussian distribution near the centerline of the plume emanating from an emission source, assuming uniform and continuous source strength, with mass conservation throughout the dispersion process. According to the national standard “Technical methods for making local emission standards of air pollutants” (GB/T 3840-1991) [16], the Gaussian plume model is recommended for use in the assessment of atmospheric pollutant dispersion under routine conditions. The model calculations are given in Equations (3)–(5) [16]:

(3) $$EPC=\frac{ER}{2\pi U_{air}\sigma_{y}\sigma_{z}}\times \exp(-\frac{{y_{air}}^{2}}{2\sigma_{y}^{2}})\times \{\exp[-\frac{({Z_{air}-\delta_{air})}^{2}}{2\sigma_{z}^{2}}]+\exp[-\frac{({Z_{air}+\delta_{air})}^{2}}{2\sigma_{z}^{2}}]\}$$

(4) $$\sigma_{y}=\gamma_{1}x^{\alpha_{1}}$$

(5) $$\sigma_{Z}=\gamma_{2}x^{\alpha_{2}}$$

where EPC represents the exposure concentration of a pollutant at a certain distance from the source area, measured in mg/m^3^; $\sigma_{y}$ is the lateral atmospheric dispersion coefficient in m; $\sigma_{z}$ is the vertical atmospheric dispersion coefficient, also in m; $y_{air}$ is the lateral distance to the centerline of the atmospheric pollution plume, measured in m; $\delta_{air}$ is the breathing zone height in m; x is the distance from the residential area to the pollution source area in m; $\alpha_{1}$ is the regression exponent for the lateral dispersion parameter; $\alpha_{2}$ is the regression exponent for the vertical dispersion parameter; $\gamma_{1}$ is the regression coefficient for the lateral dispersion parameter; and $\gamma_{2}$ is the regression coefficient for the vertical dispersion parameter. The values of $\alpha_{1}$, $\alpha_{2}$, $\gamma_{1}$, and $\gamma_{2}$ are primarily determined according to the corresponding atmospheric stability classes specified in GB/T 3840-1991 [16].

Among these parameters, the bulk density of solid waste, particle density, moisture content, and toxic substance concentration were determined through actual measurements. The dispersion of pollutants in the atmosphere is closely linked to atmospheric stability. Commonly used methods for classifying atmospheric stability include the Pasquill stability classification scheme and the approach recommended by the International Atomic Energy Agency [17,18]. China currently adopts the Pasquill stability classification scheme. Atmospheric stability is categorized into six grades: very unstable (A), unstable (B), weakly unstable (C), neutral (D), moderately stable (E), and stable (F).

### 2.4. Characterization of Odor Concentration and Intensity

According to the “Standard for emission of malodorous pollutants” (GB 14554-1993) [19], the odor concentration is defined as the dilution factor when an odor sample is continuously diluted with odorless air until the threshold of odor perception by an olfactometer is reached. The calculations can be performed using Equations (6) and (7) [19]:

(6) $${OAV}_{i}=\frac{C_{i}}{C_{Ti}}$$

(7) $$OAV=\sum {OAV}_{i}$$

where ${OAV}_{i}$ denotes the odor activity value of pollutant *i* in the mixed gas, a dimensionless quantity; $C_{i}$ represents the mass concentration of pollutant *i*, measured in mg/m^3^’; $C_{Ti}$ is the olfactory threshold concentration of pollutant *i*, measured in mg/m^3^; and OAV is the total odor activity value of the mixed gas, a dimensionless measure.

Odor intensity is a direct reflection of the human perception of malodorous pollution, providing a simple and intuitive indication of the degree of olfactory stimulation experienced by individuals. To predict odor intensity for single components, various models have been established, including Weber–Fechner’s law, power-law models, and linear models [20,21]. Among these, Weber–Fechner’s law has been widely applied due to its high predictive accuracy. This model posits that the perceived magnitude of an odor is proportional to the logarithm of the stimulus intensity affecting the olfactory senses. The choice of this model is further justified by its alignment with the actual human olfactory perception mechanism, thereby offering more accurate results in assessing odor intensity. This relationship can be represented by Equation (8) [19]:

(8) $${OI}_{i}=k_{i}lg\left({OAV}_{i}\right)+B_{i}$$

where ${OI}_{i}$ represents the odor intensity of pollutant *i* in the mixed gas, a dimensionless quantity, while $k_{i}$ and $B_{i}$ are fitting parameters specific to pollutant *i*.

Regarding the classification of odor intensity (${OI}_{i})$, China currently employs a six-level system. Level 0 indicates no odor, Level 1 denotes slight odor perception, Level 2 refers to weak odor sensation, Level 3 describes noticeable odor, Level 4 signifies strong odor, and Level 5 represents intensely unbearable odor. This odor intensity (${OI}_{i})$ classification provides sensory descriptions for the different levels of olfactory impact.

### 2.5. Uncertainty and Sensitivity Characterization

The Monte Carlo method was used to assess parameter uncertainty’s impact on the results [7]. Risk was evaluated in two ways: first, by calculating the probability of exceeding standards at various exposure points using cumulative frequency distributions and second, by analyzing exceedance multiples based on the 95th percentile concentration. Key uncertainties stemmed from source emission strengths and pollutant dispersion parameters. The parameters for atmospheric stability classification and the relevant parameters of the Gaussian model were primarily based on GB/T 3840-1991 [16]. Additionally, parameters such as the H and Koc of the pollutants were mainly referenced from HJ 25.3-2019 [5].

## 3. Results and Discussion

### 3.1. Volatile Organic Pollutant Characteristics of Solid Waste

The analysis of toxic substances revealed the presence of various benzene derivatives and polycyclic aromatic hydrocarbons at different concentrations. Based on comparisons with odorant inventories from China, Japan, and the United States and considering both detection frequency and concentration levels, ethylbenzene (ET), toluene (TO), styrene (ST), and m/p-xylene (M/p-X) were identified as priority pollutants for further assessment. Given their influence on odor risk calculations, toxic substance contents were determined using uncertain parameter methods.

**Figure 1:**
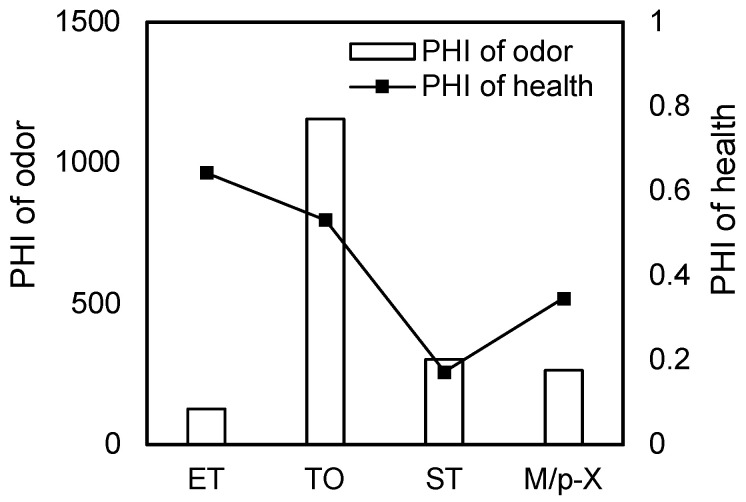
Potential hazard index of contaminant components.

The Potential Hazard Index (PHI) is defined as the ratio of the source strength concentration of pollutants to their regulatory limits [17,22]. It serves as an indicator of the potential health risks and odor hazards for residents located downwind of the emission source. To assess the human health risk (PHI) associated with VOCs, the average pollutant concentrations were compared with their corresponding risk screening values specified in GB 36600-2018 [23]. Exceeding these screening values generally indicates a potential environmental risk. Additionally, the ratio of the average pore gas pollutant concentration to its respective odor threshold was calculated to represent the potential odor hazard index, as illustrated in Figure 1.

As shown in Figure 1, the PHI values for health risks associated with toluene, styrene, and m/p-xylene ranged from 0.17 to 0.64, all of which were below the critical threshold of 1. Ethylbenzene had the highest PHI for health risks, with a mean concentration of 18 mg/kg, which was still lower than the risk screening value of 28 mg/kg. Consequently, the short-term potential health hazards posed by VOCs in solid waste to the surrounding population were relatively minor. However, the odor risk PHI values for these four pollutants ranged from 127 to 1156, indicating that the VOC concentrations in pore gases far exceeded their respective olfactory thresholds. The olfactory thresholds for ethylbenzene, toluene, styrene, and m/p-xylene are 0.085 mg/m^3^, 0.403 mg/m^3^, 0.158 mg/m^3^, and 0.431 mg/m^3^, respectively. Toluene had the highest odor PHI value at 1156, which was 2177 times greater than its health risk PHI value. Similarly, the odor PHI values for ethylbenzene, styrene, and m/p-xylene were 198, 1757, and 762 times greater than their respective health-risk PHI values. Therefore, although these four typical pollutants pose minimal short-term health risks to the local population, overly hasty emergency clean-up operations could lead to the dispersion of VOCs, potentially causing odor concentrations in residential areas to exceed acceptable limits and generating unwarranted public concern.

### 3.2. Odor Exposure Risk and Its Evolution During Emergency Excavation

**Figure 2:**
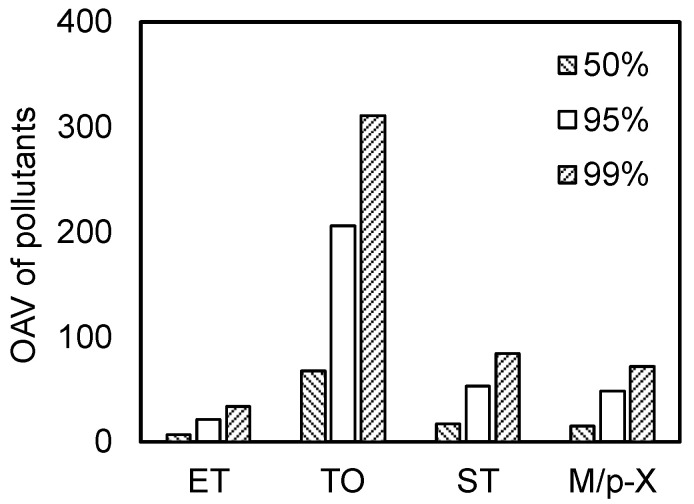
Comparison of odor concentrations for pollutants at the 50th, 95th, and 99th percentiles.

The upper control limit for concentration, a critical parameter in monitoring the risks associated with pollutants, is defined internationally as an estimate of the exposure point concentration. In domestic risk assessments, the upper control limit is often set as the median or maximum concentration of the pollutant [24,25]. To address this, in this study, odor concentrations were compared at different cumulative frequencies, as shown in Figure 2. The figure demonstrates that there was a considerable disparity in odor concentrations for the same pollutant at different cumulative frequencies. Taking toluene as an example, the odor concentrations at cumulative frequencies of 50%, 95%, and 99% were 67, 206, and 310, respectively. The odor concentration at the 50th percentile, reflecting average levels, was only 32.5% of that at the 95th percentile. The exposure concentration at the 99th percentile, which was indicative of extremely adverse scenarios, was 1.5 times greater than that at the 95th percentile. Clearly, selecting the median as the exposure concentration would underestimate the odor concentration, leading to a misjudgment of risks. Conversely, opting for the maximum value would result in overly conservative risk assessments, potentially delaying emergency clean-up operations for VOC-contaminated waste and increasing the risks of soil and groundwater contamination by solid waste. Therefore, we opted to calculate the odor concentrations using the upper bound of the 95% confidence interval for exposure concentrations.

**Figure 3:**
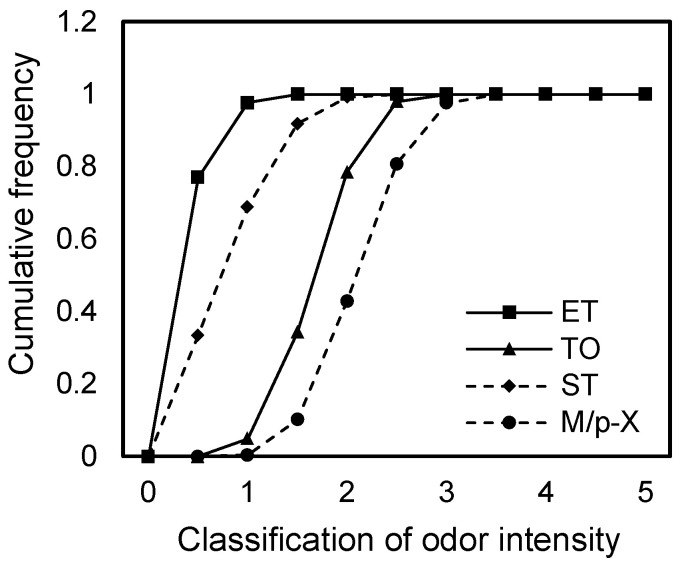
Cumulative distribution curve of odor intensity.

As shown in Figure 3, the cumulative frequency of odor intensity for pollutant components within a residential area 50 m downwind of the source was obtained using the Monte Carlo method under conditions of uncertainty.

The odor intensity of ethylbenzene ranged from −0.06 to 1.96, with a 29.6% probability of exceeding Level 1. For toluene, the odor intensity ranged from 0.65 to 2.75, with an 85% probability of exceeding Level 1 and 18.2% probability of exceeding Level 2. Styrene showed odor intensities between 0.14 and 2.99, with 66.3% and 17.6% probabilities of exceeding Levels 1 and 2, respectively. m/p-Xylene had the highest range, from 0.87 to 3.24, with a 92.5% probability of exceeding Level 1, a 41.3% probability of exceeding Level 2, and a 2.8% probability of exceeding Level 3. The order of odor intensity levels was as follows: m/p-xylene > toluene > styrene > ethylbenzene. However, this differed from the odor PHI ranking, which was as follows: toluene > m/p-xylene > styrene > ethylbenzene. This discrepancy was mainly due to the fact that exposure point concentrations are influenced not only by source strength but also by atmospheric dilution and attenuation. Comparison of the diffusion coefficients of toluene and m/p-xylene revealed that toluene exhibits stronger dilution and dispersion capabilities, resulting in lower concentrations at exposure points despite its higher source strength.

### 3.3. Analysis of the Odor’s Impact Area

**Figure 4:**
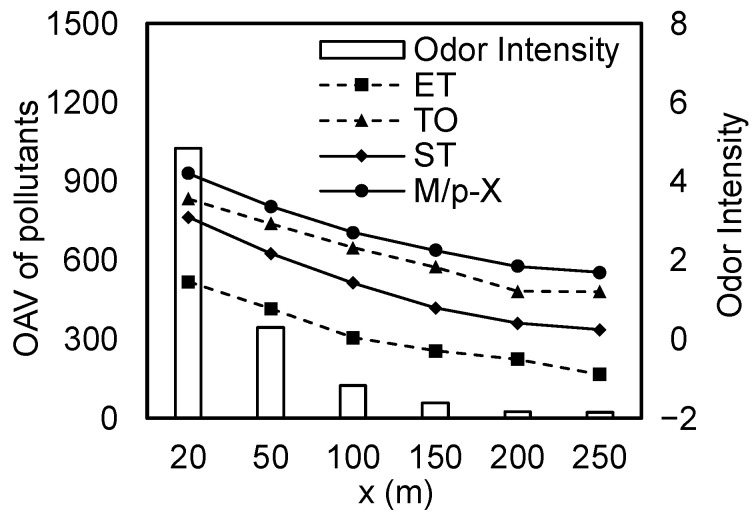
Variations in odor intensity and concentration with distance.

After being released from the mechanically excavated area, VOCs undergo atmospheric transport and diffusion, entering the downwind residential area. The variations in the odor concentration and intensity of each pollutant with distances of 20, 50, 100, 150, 200, and 250 m are shown in Figure 4.

As shown in Figure 4, the odor concentration at 20 m exceeded 1000, with VOC concentrations over 1000 times greater than the odor threshold, indicating a significant risk of odor nuisance. As the distance increased to 50 m, the odor concentration fell significantly to 346, and at 250 m, it further decreased to around 20, which was barely perceptible. Therefore, ensuring sufficient buffer distances can effectively mitigate odor risks. This finding is aligned with the conclusions of Zhang et al. [4], who conducted an odor risk assessment for the excavation of VOC-contaminated soil and found that the distance between sensitive targets and contaminated sites had the most significant impact on odor concentrations at the exposure points.

Comparing the trends of odor intensity with distance for the four pollutants, it was evident that the odor intensity of all four pollutants decreased with increasing distance, and the trends of decreasing odor intensity levels were generally consistent. The odor intensity levels of ethylbenzene, toluene, styrene, and m/p-xylene ranged from −0.89 to 1.46, 1.21 to 3.56, 0.25 to 3.10, and 1.70 to 4.20, respectively. Notably, when the distance increased from 20 to 50 m, the odor intensity of ethylbenzene decreased to less than 1, making it barely perceptible by the olfactory sense. At 50 m, the odor intensity level of styrene was 2.2, indicating a faint odor, which decreased to less than 1 at 150 m. Toluene and m/p-xylene had odor intensity levels of 2.9 and 3.4, respectively, at 50 m, and their odors were distinctly perceived at the exposure points. When the distance increased to 250 m, the odor intensity levels of toluene and m/p-xylene decreased to 1.2 and 1.7, respectively, but they were still faintly perceivable. Yan et al. [3] studied the impact of excavation disturbances on the distribution of VOC pollutants in contaminated sites and found that the soil’s organic matter content can affect the release of volatile compounds, with increased soil organic matter content reducing the range of the odor’s impact. Therefore, during emergency excavation and clean-up, the use of adsorbent materials such as activated carbon can be considered for reducing the odor pollution intensity at downwind exposure points.

### 3.4. The Influence of Meteorological Conditions on Odor Risk at the Exposure Points

**Figure 5:**
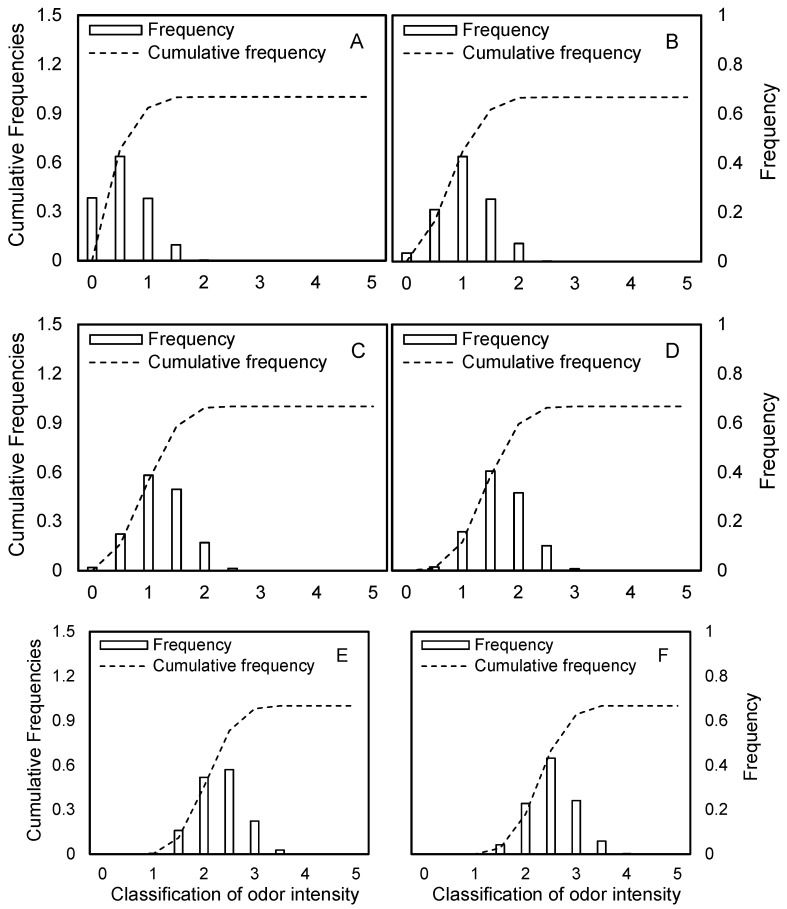
Probability distribution of odor intensity for m-/p-xylene under different odor intensity levels. Atmospheric stability classes: A (very unstable) to F (stable).

Focusing on m/p-xylene, which posed the highest odor risk, the frequency distribution and cumulative probability of atmospheric odor intensity were analyzed at a residential area located 50 m downwind under varying meteorological conditions (Figure 5). As atmospheric stability increased from very unstable (Class A) to stable (Class F), the dispersion conditions deteriorated, leading to a progressive rise in odor intensity at the exposure point. Under very unstable (A) conditions, the peak odor intensity was 0.5, with no occurrences of odor grades exceeding 2.5, and the 95th percentile value was 1.51. For unstable (B) conditions, the peak intensity rose to 1.0, with the 95th percentile at 1.84, still showing no exceedance of grade 2.5. Under weakly unstable (C) conditions, the peak reached 1.5, with 4.6% of cases exceeding grade 2.5, and the 95th percentile was 2.38. In neutral (D) conditions, the peak remained at 1.5, but the cumulative frequency of odor concentrations above 2.5 increased to 10.2%, with the 95th percentile at 2.78. When atmospheric stability was moderately stable (E), the peak odor intensity increased to 2.0, with 44.2% of cases exceeding grade 2.5, and the 95th percentile reached 3.2. Finally, under stable (F) conditions, the peak odor intensity was 2.5, with 70.8% of cases exceeding grade 2.5, and the 95th percentile odor intensity was 3.5.

These results clearly demonstrate a strong correlation between increasing atmospheric stability and elevated odor risk, highlighting the significant influence of meteorological conditions on odor dispersion and exposure levels. The substantial differences in odor intensity across stability classes underscore the importance of incorporating real-time or scenario-based meteorological data into odor risk assessments, particularly during emergency responses involving VOC-contaminated waste. Relying solely on average or steady-state conditions may lead to an underestimation of peak exposure risks, potentially compromising public health and community acceptance of remediation efforts. Therefore, adaptive risk management strategies—such as adjusting excavation schedules or enhancing control measures based on prevailing weather conditions—are essential for minimizing odor-related impacts and ensuring timely and effective cleanup operations.

**Figure 6:**
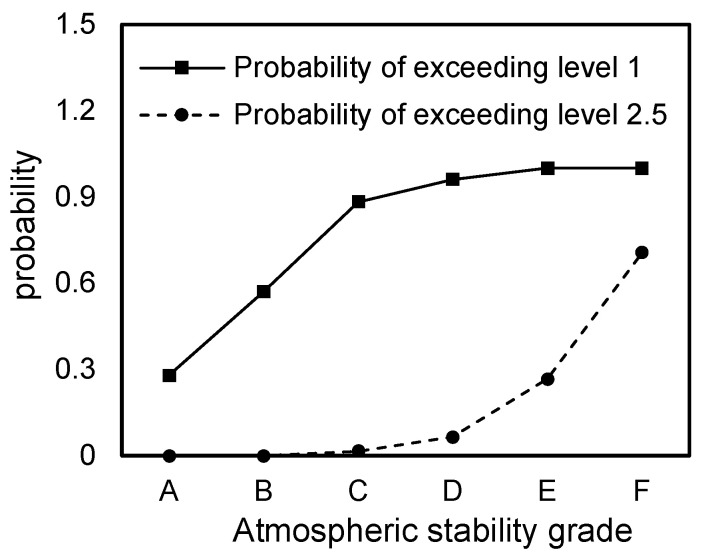
Exceedance probabilities of odor intensity at exposure points under different meteoro-logical stability classes.

When the odor intensity at the exposure point exceeded Level 1, residents could perceive the odor. When it surpassed Level 2.5, it was generally considered that the atmosphere was polluted by an odor, and therefore measures were required to reduce pollution. The probabilities of the m-/p-xylene odor intensity exceeding Levels 1 and 2.5 were calculated and are presented in Figure 6. As shown in Figure 6, under strongly unstable atmospheric stability (A), the probabilities of the odor intensity exceeding Levels 1 and 2.5 were 38.12% and 0%, respectively. For unstable conditions (B), these probabilities were 59.78% and 0%. In weakly unstable conditions (C), the probabilities increased to 89.02% and 1.61%, respectively. Under neutral atmospheric stability (D) the probabilities were 97.38% and 9.62%, respectively. Under moderately stable conditions (E), the probabilities were 100.00% and 46.08%, respectively. Finally, under more stable conditions (F), the probabilities were 100.00% and 78.59%, respectively.

Despite the significant progress made in assessing odor risks during the emergency cleanup of VOC-contaminated waste, this study has are several limitations. The uncertainties in model parameters, particularly concerning meteorological conditions and soil properties, significantly impact the results. Although the Monte Carlo method was employed to quantify these uncertainties, further refinement of parameter estimation is needed for more accurate predictions in practical applications. Additionally, advancements in sensor technology for real-time VOC emission monitoring can be integrated into existing risk assessment models to provide more dynamic and precise risk management strategies. This integration aims to enhance prediction accuracy and effectively address potential odor risks.

## 4. Conclusions

This study comprehensively investigated odor risks associated with the emergency cleanup of VOC-contaminated waste and proposed corresponding management strategies. Through detailed sample analysis and model calculations, the emission, migration, and dispersion characteristics of pollutants under different meteorological conditions were clarified, and their impacts on odor intensity levels in residential areas were quantified. For instance, at 50 m from the pollution source, the odor intensity level of toluene ranged from 1.21 to 3.56, with an 85% probability of exceeding Level 1 and an 18.2% probability of exceeding Level 2. This study also revealed that changes in atmospheric stability significantly influenced odor risk—when shifting from very unstable to stable conditions, odor intensity levels rose from 0.5 to 2.5. Under moderately stable conditions (Class E), the odor intensity of m/p-xylene reached Level 2, with a 44.2% probability of exceeding Level 2.5. These findings highlight the importance of selecting appropriate buffer distances (at least 50 m) and using odor-reduction materials during emergency cleanup operations. Although the Monte Carlo method was employed to address parameter uncertainty, further refinement of key parameters is required for improved prediction accuracy in practical applications. Future research should focus on optimizing model parameters, expanding the range of VOCs considered, and exploring broader application scenarios to enhance the effectiveness and adaptability of odor risk-management strategies.

## Data Availability

The data that support the findings of this study are available from the corresponding author upon reasonable request.

## References

[1] Zhang C., Huang L., Tu H., Wang D., Wu X., Zhu X., Zhao Z., Zhang H. (2024). Research on Application of Big Data in the Screening of Environmental Illegal and Criminal Clues of Hazardous Waste. J. Ecol. Rural Environ..

[2] Jia H., Gao S., Duan Y., Fu Q., Che X., Xu H., Wang Z., Cheng J. (2021). Investigation of Health Risk Assessment and Odor Pollution of Volatile Organic Compounds from Industrial Activities in the Yangtze River Delta Region, China. Ecotoxicol. Environ. Saf..

[3] Yan Y., Xue N., Zhou L., Cong X., Li F., Yang B., Liu B. (2014). Distribution Characteristics of HCHs and DDTs during Excavation of a Contaminated Site. J. Environ. Eng. Technol..

[4] Zhang M., Pan L., Wang Z., Guo M., Wu X. (2023). Analysis of the Limits of Air Pollutants at Enterprise Boundary Based on Ambient Multimedia Environmental Goals Estimation. J. Environ. Eng. Technol..

[5] (2019). Technical Guidelines for Risk Assessment of Soil Contamination of Land for Construction.

[6] Su Y., Cheng W., Li Y., Sun Y., Wang X., Hou H., Wang J. (2023). Study on Acceptable Levels of Volatile Organic Compounds in Air and Soil Gas in Contaminated Sites Based on Guideline Model. Environ. Prot. Sci..

[7] Li R., Yuan J., Li X., Zhao S., Lu W., Wang H., Zhao Y. (2023). Health Risk Assessment of Volatile Organic Compounds (VOCs) Emitted from Landfill Working Surface via Dispersion Simulation Enhanced by Probability Analysis. Environ. Pollut..

[8] Seo J.H., Kim P.-G., Choi Y.-H., Shin W., Sochichiu S., Khoshakhlagh A.H., Kwon J.-H. (2025). Evaluation of Personal Exposure to Volatile Organic Chemicals (VOCs) in Small-Scale Dry-Cleaning Facilities Using Passive Sampling. Atmos. Environ..

[9] Gallego E., Perales J.F., Aguasca N., Domínguez R. (2024). Determination of Emission Factors from a Landfill through an Inverse Methodology: Experimental Determination of Ambient Air Concentrations and Use of Numerical Modelling. Environ. Pollut..

[10] (2019). Technical Specifications on Identification for Hazardous Waste.

[11] (2011). Soil and Sediment-Determination of Volatile Organic Compounds—Purge and Trap Gas Chromatography/Mass Spectrometry Method.

[12] Ge M., Zheng Y., Zhu Y., Ge J., Zhang Q. (2023). Effects of Air Exchange Rate on VOCs and Odor Emission from PVC Veneered Plywood Used in Indoor Built Environment. Coatings.

[13] Zhang K., Chang S., Fu Q., Sun X., Fan Y., Zhang M., Tu X., Qadeer A. (2021). Occurrence and Risk Assessment of Volatile Organic Compounds in Multiple Drinking Water Sources in the Yangtze River Delta Region, China. Ecotoxicol. Environ. Saf..

[14] Snoun H., Krichen M., Chérif H. (2023). A Comprehensive Review of Gaussian Atmospheric Dispersion Models: Current Usage and Future Perspectives. Euro-Mediterr. J. Environ. Integr..

[15] Khan S., Hassan Q. (2021). Review of Developments in Air Quality Modelling and Air Quality Dispersion Models. J. Environ. Eng. Sci..

[16] (1991). Technical Methods for Making Local Emission Standards of Air Pollutants.

[17] Ma L., Zhao R., Li J., Yang Q., Liu Y. (2024). Release Characteristics and Risk Assessment of Volatile Sulfur Compounds in a Municipal Wastewater Treatment Plant with Odor Collection Device. J. Environ. Manag..

[18] Pei Y., Liu N., Liu S., Guan H., Guo Z., Li Q., Han W., Cai H. (2022). Investigation of Odor Emissions from Coating Products: Key Factors and Key Odorants. Front. Environ. Sci..

[19] (1993). Emission Standards for Odor Pollutants.

[20] Zhou Y., Vitko T.G., Suffet I.H. (2023). A New Method for Evaluating Nuisance of Odorants by Chemical and Sensory Analyses and the Assessing of Masked Odors by Olfactometry. Sci. Total Environ..

[21] Yin Z., Bader T., Lee L.F., McDaniels R., Suffet I.H. (2025). Training a Regulatory Team to Use the Odor Profile Method for Evaluation of Atmospheric Malodors. Atmosphere.

[22] Fang W., Huang Y., Ding Y., Qi G., Liu Y., Bi J. (2022). Health Risks of Odorous Compounds during the Whole Process of Municipal Solid Waste Collection and Treatment in China. Environ. Int..

[23] (2018). Soil Environmental Quality Risk Control Standard for Soil Contamination of Development Land.

[24] Jia J., Zhang B., Zhang S., Zhang F., Ming H., Yu T., Yang Q., Zhang D. (2024). Appropriate Control Measure Design by Rapidly Identifying Risk Areas of Volatile Organic Compounds during the Remediation Excavation at an Organic Contaminated Site. Environ. Geochem. Health.

[25] Wei G., Yang X. (2023). Effect of Temperature on VOC Emissions and Odor from Vehicle Carpet. Build. Environ..

